# Optimizing genetic testing strategy for suspected attenuated adenomatous polyposis: effective solutions in public health systems

**DOI:** 10.1007/s12094-024-03811-y

**Published:** 2024-12-11

**Authors:** Natalia García-Simón, Fátima Valentín, Ana Royuela, Beatriz Hidalgo-Calero, Ricardo Blázquez-Martín, Montserrat de-Miguel-Reyes, José María Sánchez-Zapardiel, Luisa Adán-Merino, Alejandro Rodríguez-Festa, Patricia Gallego-Gil, Pilar Mediavilla-Medel, Laura Quiñonero-Moreno, Lourdes Gutiérrez, Alberto Herreros-de-Tejada, Antonio Sánchez, Mariano Provencio, Atocha Romero

**Affiliations:** 1 Hereditary Cancer Unit, Medical Oncology Department, Puerta de Hierro University Hospital, Majadahonda, 28222 Madrid, Spain; 2 Gastroenterology Department, Biomedical Research Institute (IDIPHISA), Puerta de Hierro University Hospital, Majadahonda, Madrid, Spain; 3 Biostatistics Unit, Puerta de Hierro Biomedical Research Institute (IDIPHISA), CIBERESP, ISCIII. Majadahonda, Madrid, Spain; 4 Hereditary Cancer Laboratory, 12 de Octubre University Hospital, Madrid, Spain; 5 Gastroenterology Department, Infanta Leonor University Hospital, Madrid, Spain

**Keywords:** Hereditary attenuated adenomatous polyposis, Age, Adenomas, Genetic testing, *APC*, *MUTYH*

## Abstract

**Background:**

*APC* and *MUTYH* genes are key in hereditary attenuated adenomatous polyposis syndromes. Guidelines recommend genetic testing based on polyp count, often overlooking age despite its impact on polyp prevalence.

**Aim:**

To enhance genetic testing strategies for suspected attenuated adenomatous polyposis by combining polyp count and age in a probability calculator.

**Methods:**

Retrospective study of adult patients referred to NGS genetic testing for suspected attenuated adenomatous polyposis (accumulated history of < 100 adenomas) (discovery cohort, N = 138). Data included age, adenoma count, and test results. A multivariable logistic regression model was developed to associate positive genetic test results with age and adenoma count. The model was externally validated with 259 patients from two tertiary hospitals in our region (validation cohort, N = 259).

**Results:**

In the discovery cohort, 13 (9.4%) patients had pathogenic mutations, being younger (OR:0.91, 95%CI 0.86–0.96) and having more adenomas (OR:1.08, 95%CI 1.04–1.13) compared to negative cases. The logistic regression model combining age and polyp count demonstrated an AUC of 0.92. Using a cutoff probability of 3.5%, the model achieved 100% sensitivity and 58% specificity in identifying positive cases. In the external validation, the model accurately predicted 14 out of 16 positive cases (88%). The remaining two positive cases were a patient with an *AXIN2* mutation in heterozygosis, and a patient with a *NTHL1* mutation in homozygosis. Performance evaluation of both hospitals yielded AUC values of 0.77 and 0.90.

**Conclusions:**

Older individuals with fewer polyps are less likely have hereditary syndromes. Including age in genetic testing criteria can enhance patient selection and cost-effectiveness.

**Supplementary Information:**

The online version contains supplementary material available at 10.1007/s12094-024-03811-y.

## Introduction

Hereditary polyposis syndromes are known to be accountable for about 2–3% of all cases of colorectal cancer (CRC) [1, 2]. The most common polyposis syndromes are familial adenomatous polyposis (FAP) (OMIM #175,100), attenuated FAP (AFAP) (OMIM #175,100), and *MUTYH*-associated polyposis (MAP) (OMIM #608,456), while other syndromes such as hamartomatous polyposis are less frequent [3]. The main genes associated with hereditary adenomatous polyposis syndromes are *APC* (OMIM #611,731) gene, for FAP and AFAP, and *MUTYH* (OMIM #604,933) gene, for MAP.

For suspected patients, guidelines recommend offering genetic testing based on the number of polyps, with a threshold of more than 100 adenomatous polyps for FAP, and more than 10 or 20 adenomatous polyps (depending on the guideline) for AFAP and MAP [4–8]. However, since polyps are not only caused by mutations in polyposis genes but are also intrinsic to age, the older the patient is, the more likely it is to detect polyps, lowering the probability of being a case of hereditary syndrome, especially when the polyp burden is low. Therefore, despite the selection of patients, germline multigene testing continues to have a high demand in laboratories, which decreases the rate of mutation detection, making these studies low cost-effective. Stanich et al*.* [9] demonstrated that, on the one hand, the prevalence of mutations in adenomatous polyposis syndromes genes (*APC* and *MUTYH*) increases with the number of polyps developed, and on the other hand, older populations have a lower prevalence of finding significant mutations in these genes.

Consequently, age should also be included as a criterion for referring to genetic testing, helping the selection of patients, although very few guidelines include it. In this paper, we aim to improve genetic testing performance in suspected attenuated adenomatous polyposis by establishing a probability calculator based on the number of polyps and age upon which recommend referring to genetic testing.

## Methods

### Subjects

We conducted a retrospective analysis of patients aged 18 years and older referred for genetic testing at Puerta de Hierro Hospital for suspected attenuated adenomatous polyposis (AFAP or MAP) between 2015 and 2023 (N = 138). Suspicion was based on a history of 10 to 100 adenomatous polyps, following the Community of Madrid (CAM) guidelines [10]. Patients with two or more hamartomatous polyps were excluded from the study as this suggests hamartomatous polyposis [4]. The study received approval from the ethics committee of Puerta de Hierro Hospital (internal code: PI_48/24). Pre-test genetic counseling was conducted, and clinical consent for genetic testing was obtained. Written informed consent for data publication was also obtained from patients.

Only pathogenic (P) (class 5) and likely pathogenic (LP) (class 4) variants in *APC* and *MUTYH* genes were considered positive cases. Being a recessive gene, *MUTYH* variants were classified as positive only if found in homozygosity or compound heterozygosity. Negative cases included no variants detected, benign (class 1) and probably benign (class 2) variants, variants of uncertian significance (class 3), or monoallelic *MUTYH* variants.

### Genetic testing

At Puerta de Hierro Hospital, germline DNA was extracted from peripheral blood using the Maxwell RSC whole blood DNA kit (Promega). Genetic testing was performed by massive sequencing (NGS) on a MiSeq sequencer (Illumina) using the Hereditary Cancer Solution (HCS) kit (Sophia Genetics) and following the manufacturer’s instructions. The panel included *APC* and *MUTYH* as relevant genes associated with adenomatous polyposis. Bioinformatic analysis was performed using the Sophia DDM‐V4 (Sophia Genetics) data analysis platform. Relevant SNPs and indels were confirmed by Sanger sequencing. The reference sequences used to name variants were NM_001128425.2 for *MUTYH* and NM_000038.6 for *APC.*

### Age and number of polyps

Age refers to the age at genetic testing. Number of polyps refers to the total accumulated polyps until genetic testing.

Polyps were histologically classified into adenomatous (tubular, tubulovillous and villous), and non-adenomatous (hyperplastic and serrated polyps) groups. There were some reports that classified resected polyps just as “adenomatous” without sub-classification. They are here reported as “not classified” adenomatous polyps and were only considered in the adenomatous vs non-adenomatous polyps’ comparison and not in the subtype comparison.

### External validation

Two independent cohorts (N = 259) were used for validation: 12 de Octubre University Hospital (n = 162) and Infanta Leonor University Hospital (n = 97).

At 12 de Octubre University Hospital, extracted DNA from whole blood using the Maxwell RSC Whole Blood kit (Promega). The Custom Hereditary Cancer Solution (CHCS) kit (Sophia Genetics) was employed for genetic testing, and software analysis was conducted using Sophia DDM‐V4 (Sophia Genetics). Genes included in the sequencing kit were *APC, MUTYH, POLE, POLD1, AXN2* and *NTHL1*. Any pathogenic or LP variants identified through massive sequencing were subsequently validated via Sanger sequencing.

Infanta Leonor Hospital utilized the QIAamp Blood DNA kit (QIAcube) for the extraction and purification of DNA from peripheral blood. Genetic testing was conducted by NGS on a MiSeq (Illumina) using the SureSelect QXT Target Enrichment (Agilent) kit for the coding region and flanking zones of the analyzed genes (*APC, MUTYH, POLE, POLD1, NTHL1, MSH3*). The bioinformatic analysis was carried out using custom-designed analysis pipelines, assisted by the SureCall and Alissa Interpreter software (Agilent). Sanger sequencing was employed to confirm relevant SNPs.

### Statistics

The Shapiro–Wilk test assessed normality. Non-normally distributed quantitative variables were presented as median along with the 25th (P25) and 75th (P75) percentiles. For nonparametric comparisons, the Chi square test and Mann–Whitney test were used for categorical and quantitative variables respectively. Multivariable logistic regression (logit) established the association between having a positive genetic test result (dependent variable) and the age and polyps count. Internal validation used the bsvalidation command in Stata [11]. This command performs an internal validation through calibration and discrimination. Resampling techniques were performed by bootstrapping, with 500 replications. To evaluate calibration, a calibration plot was generated, in which the quintiles of the observed and expected probabilities of having the event were graphically confronted. The expected/observed (E/O) ratio will equal 1, the calibration in the large (CITL) will be 0 and the slope equal to 1. Discrimination is measured by the C-statistic, which is an analog of the AUC, with values ranging from 0.5 for no discrimination to 1.0 for perfect discrimination. The Brier scale (range 0–100) was also calculated as an overall performance measure, with high values indicating predictions closer to the actual outcome. It was obtained from the Brier score: Brier scaled = 1 – Brier score / Brier max.

For the external validation, the calibration plot assessed the calibration and the AUC, the discrimination.

From the model predicted probability, we pursued an optimal cutoff point with the maximal sensitivity and developed an online calculator available at https://investigacionpuertadehierro.com/calculadora-poliposis/.

P value < 0.05 was considered statistically significant.

Statistical analysis was carried out using MedCalc Statistical Software version 11.4.2.0 program (MedCalc Software bvba, Ostend, Belgium; http://www.medcalc.org; 2018), Stata v18 (StataCorp. 2023. *Stata Statistical Software: Release 18*. College Station, TX: StataCorp LLC.).

## Results

Of the 138 patients included in the Puerta de Hierro cohort, 13 patients (9.4%) tested positive for genetic mutations. Among these, 11 patients had a P/LP variant in *MUTYH* gene: three were homozygous and eight were compound heterozygous. Two patients had a P variant in *APC* gene in heterozygosis (Supplementary Fig. 1). The most prevalent *MUTYH* mutations were c.1187G > A p.(Gly396Asp) (commonly known as G396D), and c.536A > G p.(Tyr179Cys) (commonly known as Y179C) (Table 1).

**Table 1:** Characteristics of positive group patients

Patient	Age at GT (years)	Gene	Variant	Heterozygosity	CRC (Age diagnosis)	FH CRC
Case 1	75	*MUTYH*	c.1187G > A	Homozygous	YES (57)	NO
Case 2	28	*MUTYH*	c.536A > G + c.933 + 3A > C	Compound heterozygous	NO	YES
Case 3	47	*MUTYH*	c.1012C > T + c.536A > G	Compound heterozygous	NO	YES
Case 4	44	*MUTYH*	c.1012C > T + c.536A > G	Compound heterozygous	NO	YES
Case 5	46	*MUTYH*	c.1012C > T + c.536A > G	Compound heterozygous	NO	YES
Case 6	63	*MUTYH*	c.1187G > A + c.736G > T	Compound heterozygous	YES (60)	YES
Case 7	66	*MUTYH*	c.1187G > A	Homozygous	YES (55)	YES
Case 8	77	*MUTYH*	c.1187G > A	Homozygous	NO	YES
Case 9	51	*MUTYH*	c.1187G > A + c.1227_1228dup	Compound heterozygous	NO	NO
Case 10	51	*MUTYH*	c.1187G > A + c.1101dup	Compound heterozygous	NO	NO
Case 11	44	*MUTYH*	c.1187G > A + c.1227_1228dup	Compound heterozygous	YES (44)	NO
Case 12	39	*APC*	c.697C > T	Heterozygous	NO	YES
Case 13	79	*APC*	c.423G > C	Heterozygous	NO	NO

*GT* genetic testing, *CRC* colorectal cancer, *FH* family history

Patient characteristics of positive and negative groups are shown in Table 2. There were no significant differences in sex distribution among groups. Similarly, development of CRC was similar between the two groups, with 4 (24.8%) CRC patients in the positive group and 31 (30.8%) in the negative group. Regarding family history (FH) of CRC, 43.2% of patients in the negative group had at least one family member with CRC while for the positive group, the percentage rose to 61.5%, although the difference did not reach statistical significance.

**Table 2:** Patients’ characteristic for positive and negative group

Patients’ characteristics	Positive group n = 13	Negative group n = 125	p value
Age (years) median (P25-P75)	51 (44–66)	67 (61–72)	0.012
Sex			0.51
Women, n (%)	6 (46.2)	46 (36.8)	
Men, n (%)	7 (53.8)	79 (63.2)	
Polyp type	683 (100)	3685 (100)	< 0.001
Adenomatous, n (%)	659 (96.5)	2916 (79.1)	
Non-adenomatous, n (%)	24 (3.5)	769 (20.9)	
CRC, n (%)	4 (30.8)	31 (24.8)	0.64
FH CRC, n (%)	8 (61.5)	55 (44)	0.23
Smoking, n (%)			0.004
Yes	2 (16.6)	42 (33.3)	
Former	1 (5.6)	49 (39.7)	
No	7 (55.6)	26 (20.6)	
ND	3 (22.2)	8 (6.4)	

*CRC* colorectal cancer, *FH CRC* family history of colorectal cancer, *ND* no data

Parameters that did show significant differences between the negative and positive groups were age (OR: 0.91, 95%CI 0.86–0.96, *P* = 0.012), number of adenomas (OR: 1.08, 95%CI 1.04–1.13, *P* < 0.001) and smoking status (OR: 8.17, 95%CI 1.97–33.8, *P* = 0.004).

### Age-comparison study

**Fig. 1:**
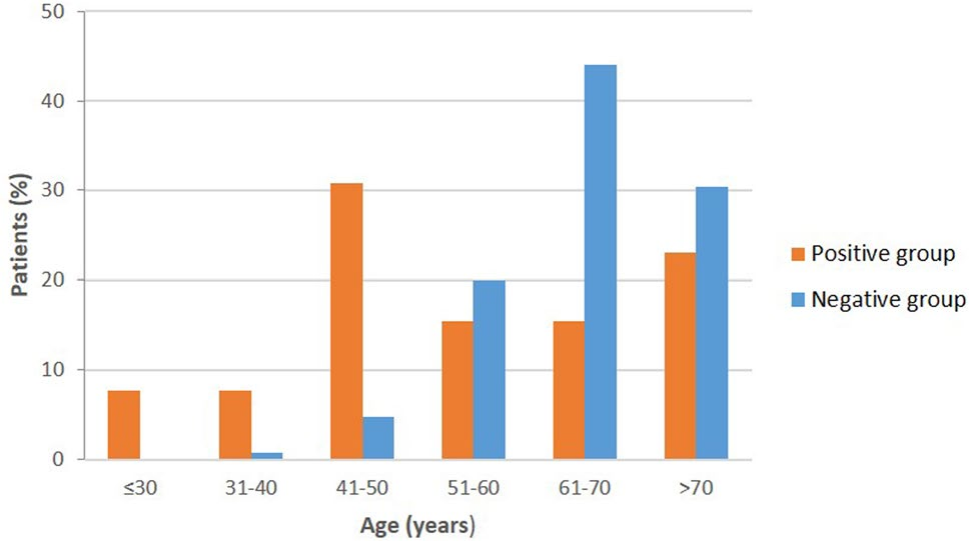
Distribution of ages (by decades of years) at which genetic testing was performed

The youngest positive case was 28 years old, and the oldest one was 79 years old. In the negative group, ages ranged from 39 to 83 years. The median age was 51 years in the positive group (P25-P75: 44–66), whereas the median age was 67 years (P25-P75: 61–72) in negative cases (OR: 0.91, 95%CI 0.86–0.96; Supplementary Fig. 2). Among negative cases, 74% were aged over 60, whereas the positive group had only five cases above that age (Fig. 1).

### Polyp comparison study

Comparison data revealed a relation between the genetic test result and the number of adenomas. Both groups developed more adenomatous polyps than non-adenomatous polyps, but overall, the positive group developed significantly more adenomas (median: 42, P25-P75: 33–74) than the negative group (median: 22, P25-P75: 16–28) (OR: 1.08, 95%CI 1.04–1.13). At the time of genetic testing, the majority of positive cases (85%) accumulated more than 30 adenomas, while only 24% of negative cases reached that threshold (Supplementary Fig. 3).

There were no significant differences according to the subtypes of polyps (Supplementary Table 1). For adenomatous subtypes, the most common one was tubular in the positive group as well as in the negative group (90.7% vs 89.9% respectively), followed far behind by tubulovillous (5.3% vs 6.1%) and villous polyp subtypes (0.5% vs 0.2%) (*P* = 0.46). For non-adenomatous polyps, the hyperplasic subtype was the most prevalent (83% vs 81%) in both groups (Supplementary Fig. 4).

### Calculator

#### Model development

**Fig. 2:**
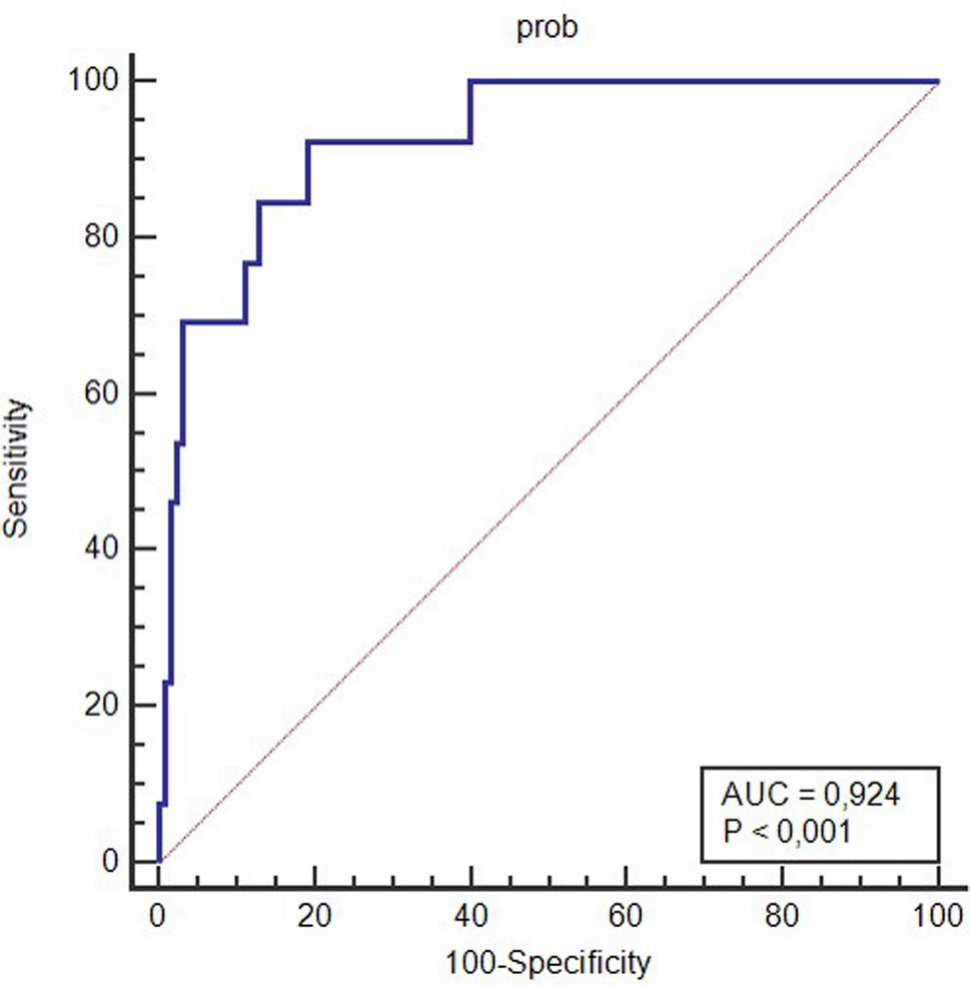
AUC for probability of having a positive genetic test

Using multivariable logistic regression, we estimated the probability of a patient having a positive genetic result based on their age and number of adenomas at genetic testing. The regression equation was: *logit (genetic test (* +*)/1- genetic test (* +*))* = *0,3822* + (−0,0814*age in years) + (0,0731*number of adenomas) ^©^ IDIPHISA, (2024), All rights reserved. Overall model performance was ranked by a Brier score of 32.6%. Calibration scores were 1 for E/O ratio, 0 (95%CI  – 0.73 to 0.73) for CITL, and 1 (95%CI 0.56–1.44) for slope. Discrimination was assessed by an AUC of 0.924 (95%CI 0.85–0.99; *P* < 0.01) (Fig. 2).

The next step was to establish a cut-off point from the predicted probability model upon which to decide whether to refer patients to genetic testing or not. The requirement set to select this point was having a 100% sensibility with maximum specificity, so the false negative rate would be 0% but minimizing the number of false positives. These criteria were fulfilled at a probability of 3.5%, with a sensibility of 100% and a specificity of 58%. Applying the model retrospectively, it was found that 74 cases meeting the polyposis criteria according to CAM recommendations had a probability of a positive genetic test below 3.5%.

#### Internal validation

For internal validation, the Brier score for overall model performance was 24.3%. Calibration results showed an E/O ratio of 0.97 (95% CI 0.57–1.38), CITL of 0.07 (95%CI  – 0.8 to 1.01), and a calibration slope of 0.89 (95%CI 0.39–1.51). C-statistic for discrimination was 0.9 (95%CI 0.78–1). After adjusting the model by bootstrapping, the OR for age was 0.93 (95%CI 0.88–0.98), and the OR for number of polyps was 1.07 (95%CI 1.03–1.1).

#### External validation

The final validation was made by using data from other centers (N = 259), located in the same geographic area. We gathered data on the number of polyps and age at the time of genetic testing, and the results of such test, classifying patients between “positive” (when genetic results revealed a P/LP mutation in genes related to polyposis) and “negative” (when no P/LP mutation related to polyposis was found). Patient characteristics from external centers closely resembled those of our own (Supplementary Table 2).

**Fig. 3:**
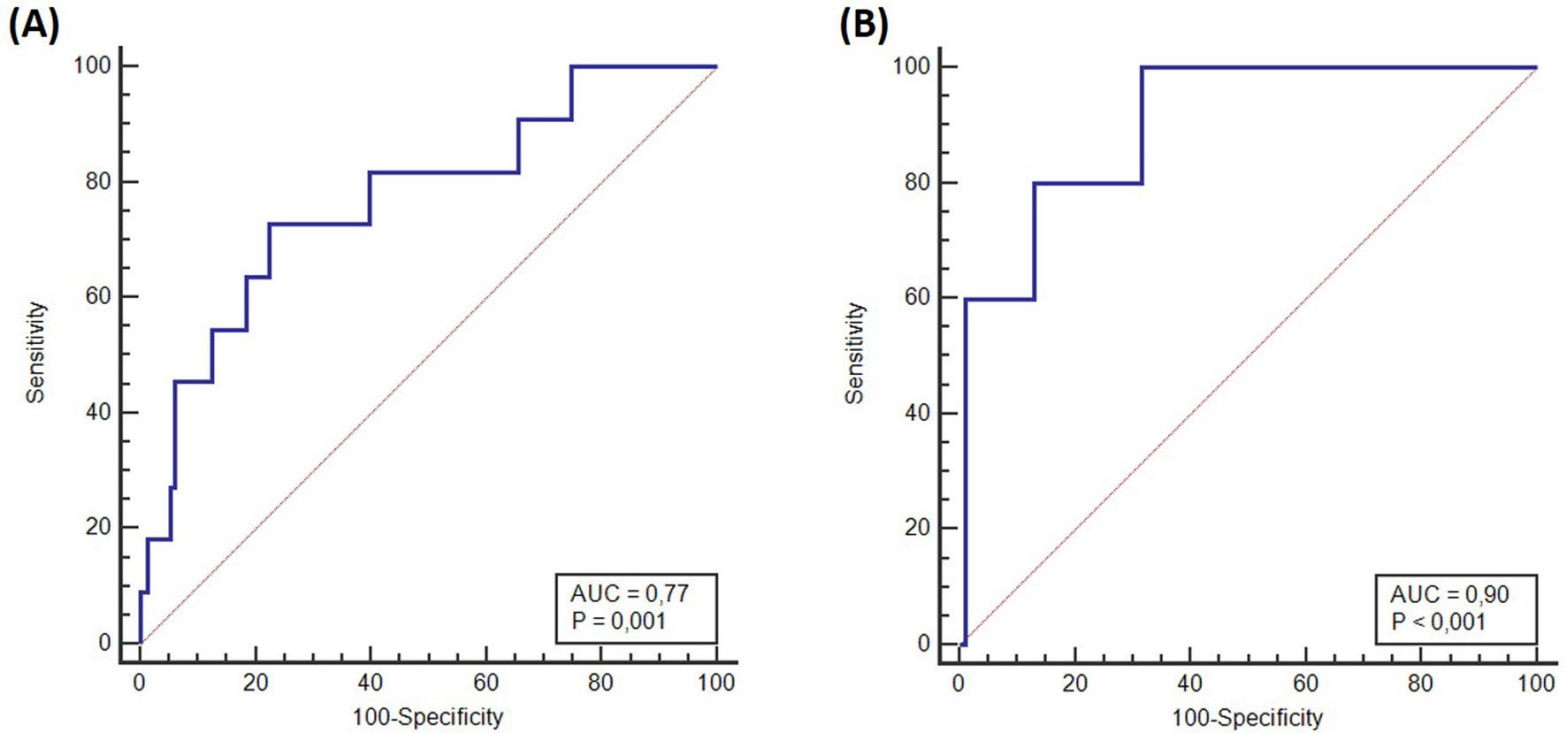
AUC resulting from applying the model to external validation databases. **A** AUC for 12 de Octubre Hospital, and **B** AUC for Infanta Leonor Hospital

At 12 de Octubre Hospital (n = 162), 11 patients were reported as positive. Of these, four cases presented biallelic *MUTYH* mutations (three homozygous and one compound heterozygous), four cases carried *APC* mutations in heterozygosis and one case presented a heterozygous *POLD1* mutation. The model correctly predicted the positive result in 9 out of these 11 cases. The remaining two positive cases were predicted as negative. One was a 67-year-old patient with 19 adenomas with the mutation c.1994dup p.(Asn666fs) in gene *AXIN2* in heterozygosis. The second case was a 71-year-old patient with 20 adenomas and the mutation c.268C > T p.(Gln90Ter) in *NTHL1* gene, in homozygosis. Calibration performance yielded a Hosmer–Lemeshow p value of 0.45 (Table 3), while the discrimination study resulted in an AUC of 0.77 (95%CI 0.61–0.93) (Fig. 3).

**Table 3:** Perform results for external validation data

Hospital	Patients			Model prediction		Performance measures	
	All n	Positive n, (%)	Negative n, (%)	TP n, (%)	TN n, (%)	Hosmer–Lemeshow p value	AUC
12 de Octubre	162	11 (6.8)	151 (93.2)	9 (81.8)	75 (49.7)	0.45	0.77
Infanta Leonor	97	5 (5.2)	92 (94.8)	5 (100)	49 (53.3)	0.38	0.90

*TP* total positives, *TN* total negatives

At the Infanta Leonor Hospital (n = 97), 92 cases were negative, and 5 cases were positive. Mutations identified in these patients included three in *MUTYH* (1 homozygous and 2 compound heterozygous), one in *APC,* and one in *PTEN*. All 5 mutated patients were accurately predicted as positive. Performance evaluation indicated a Hosmer–Lemeshow p value of 0.38 (Table 3) and an AUC of 0.90 (95%CI 0.78–1) (Fig. 3).

## Discussion

The diagnosis of Hereditary Polyposis Syndromes is crucial for patients and their families. This diagnosis begins with an oligopoliposis phenotype, and it is confirmed by genetic testing. Accurate patient selection for genetic testing is essential for public health efficiency. Since polyps can arise by either genetic mutations or aging, there is a phenotype overlap between hereditary polyposis and sporadic polyposis. The CAM’s *Prevecolon* program screens for CRC using the fecal occult blood test (FOBT) [12] leading to increased detections of asymptomatic polyposis and genetic consultations, which strain resources and yield low diagnostic returns [13, 14]. All of this emphasizes the need to implement new tools for better patient selection. To improve it, guidelines include other features to help a better distinction between genetic and sporadic polyposis [15, 16]. Nowadays age is beginning to be included too, although very few guidelines do it and there is no consensus about the cut-off limit [4, 7, 17]. Consequently, we developed a calculator based on adenoma count and patient age to better differentiate between hereditary and sporadic polyposis, aiding health professionals in selecting patients more effectively and optimizing the diagnostic yield of genetic testing.

In the Puerta de Hierro cohort, the prevalence of biallelic *MUTYH* mutations was 8% (11/138), and 1.5% (2/138) for APC mutations, in line with previous studies which ranged prevalence of these mutations in patients with oligopolyposis from 3 to 15% for MUTYH and from 2 to 9% for APC [9, 18]. Among the *MUTYH* pathogenic variants found, the most represented ones were G396D and Y179C. This is consistent with what has been previously found since most patients belonged to European population, in which these two variants are considered founder mutations [19–21].

Confrontation of other features between positive and negative group, showed no differences in sex, as described in other studies [22, 23]. Personal history of CRC and family history of CRC did not reach statistical signification between the two groups, demonstrating that the CRC risk for mutated patients in this study has been lowered due to the early diagnosis and prophylactic surgical strategies carried out (polypectomies and colectomies) that prevented developing CRC [24–26].

In terms of tobacco consumption, the negative group exhibited higher rates of smoking and former smoking compared to the positive group (OR: 8.17, 95%CI: 1.97–33.8). Tobacco is a known carcinogen and has been linked to the development of polyposis [27, 28]. Our data imply that smoking was a significant contributing factor in sporadic polyposis cases.

Adenomatous polyps represent about two-thirds of all colonic, with tubular adenomas being the most common, followed by hyperplasic polyps. Tubulovillous, villous and serrated polyps are less frequent [23]. In this study, mutated patients developed in proportion more adenomas and fewer hyperplasic polyps than not mutated cases. When comparing adenomatous and non-adenomatous subtypes separately, there were no differences in distribution. This demonstrates that genomic mutations have a greater influence on adenomatous polyp development than sporadic factors like age, but do not affect subtype distribution.

For age comparison, the median age for patients with an *APC* or biallelic *MUTYH* mutation was 51 years, significantly younger than those patients in the negative group (OR: 0.91, 95%CI: 0.86 to 0.96). Our results complement other studies that reached the same conclusion [9, 29–31]. This consolidates the use of age as a complementary criterion for referring to genetic test.

Using data on the number of adenomas, age, and genetic test results, we constructed a model to estimate the likelihood of detecting a polyposis mutation based on adenoma count and age. We established the decision point at a 3.5% probability, ensuring 100% sensitivity and nearly 60% specificity. Had this model been applied to the patients of the Puerta de Hierro Hospital cohort, more than half of the genetic tests (53.6%) could have been saved, avoiding any missed positive cases and resulting in savings of 50,000€. External validation was conducted using data from two different hospitals within the same geographic area, to minimize potential confounding factors associated with variations in patient characteristics. Performance evaluation at both centers reported a Hosmer–Lemeshow p value above 0.05, indicating no significant differences between observed and model-predicted values. In terms of discrimination, the AUC was satisfactory for both centers. However, 12 de Octubre Hospital exhibited slightly poorer performance due to two positive cases being incorrectly predicted as negative by the model. Both cases involved elderly patients (67 and 71 years) with a low number of polyps (19 and 20, respectively). Guidelines [4–8] are gradually shifting the adenoma count threshold for recommending genetic testing from 10 to 20, and those that include age, criteria are more restricted for patients over 60 years old. These two cases fell into a gray area, as one was below the 20-adenoma threshold and the other one was just in the limit with an advanced age. Consequently, depending on the guidelines applied, these two patients might not have met the requested criteria for genetic testing referral.

The model was constructed solely based on common adenomatous polyposis genes, *APC* and *MUTYH,* as positive cases. However, in recent years, additional genes such as *POLE, POLD1, AXIN2*, and *NTHL1* have been associated to adenomatous polyposis. While these genes are now included in genetic panels, they were not available at the time of testing in our center and were therefore excluded from the model.

Unlike *APC* and *MUTYH* polyposis, POLD1/POLE syndrome is characterized by the development of extraintestinal tumors, including endometrial, ovarian, brain, and pancreatic cancers [4, 32]. Patients carrying mutations in these genes exhibit a highly distinctive phenotype leading clinicians to consider POLE and POLD1 testing not only based on the polyposis phenotype but also on the broader tumor spectrum. Notably, despite the model’s limitations, we successfully detected a POLD1-positive case.

On the other hand, patients carrying AXIN2 or NTHL1 mutations have an elevated CRC risk compared to the general population, although the level of risk remains uncertain due to the low prevalence reported thus far, complicating patient management and genetic counseling. [33, 34]. In our hands, the *AXIN2* and *NTHL1* cases were wrongly predicted however they were close to the cutoff point. With additional data from subsequent colonoscopies, these cases might have been correctly classified [35].

In conclusion, hereditary polyposis syndromes present themselves at an early age and with a higher burden of adenomas than sporadic polyposis. Both features should be taken into consideration for selecting patients to refer to genetic testing. To ease the process, we developed a calculator that provides the probability of obtaining an informative genetic result based on these two characteristics. This will aid in deciding whether to proceed with genetic testing.

## Supplementary Information

Below is the link to the electronic supplementary material.

Supplementary file1 (PPTX 1086 KB)

## Data Availability

Data will be made available on request.

## References

[1] Kidambi TD, Kohli DR, Samadder NJ, et al. Hereditary polyposis syndromes. Curr Treat Options Gastroenterol. 2019;17:650–5. 31705372 10.1007/s11938-019-00251-4

[2] Byrne RM, Tsikitis VL. Colorectal polyposis and inherited colorectal cancer syndromes. Ann Gastroenterol. 2018;31:24–34. 29333064 10.20524/aog.2017.0218 PMC5759610

[3] Chen L, Ye L, Hu B. Hereditary colorectal cancer syndromes: molecular genetics and precision medicine. Biomedicines. 2022;10:3207. 36551963 10.3390/biomedicines10123207 PMC9776295

[4] National Comprehensive Cancer Network. NCCN Guidelines Insights: Genetic/Familial High-Risk Assessment: Colorectal (Version 2.2023). 2023. [cited January 16, 2024] Available from: http://www.nccn.org/professionals/physician_gls/pdf/bone.pdf

[5] Syngal S, Brand RE, Church JM, et al; American College of Gastroenterology. ACG clinical guideline: Genetic testing and management of hereditary gastrointestinal cancer syndromes. Am J Gastroenterol 2015;110:223–62 10.1038/ajg.2014.435 PMC4695986 25645574

[6] Yang J, Gurudu SR, Koptiuch C, et al. American Society for Gastrointestinal Endoscopy guideline on the role of endoscopy in familial adenomatous polyposis syndromes. Gastrointest Endosc. 2020;91:963–82. 32169282 10.1016/j.gie.2020.01.028

[7] Monahan KJ, Bradshaw N, Dolwani S, et al; Hereditary CRC guidelines eDelphi consensus group. Guidelines for the management of hereditary colorectal cancer from the British Society of Gastroenterology (BSG)/Association of Coloproctology of Great Britain and Ireland (ACPGBI)/United Kingdom Cancer Genetics Group (UKCGG). Gut 2020;69:411–44 10.1136/gutjnl-2019-319915 PMC7034349 31780574

[8] Sociedad Española de Oncología Médica (SEOM). Cáncer Hereditario. 3ª Edición. 2019. [cited January 22, 2024] Available from: https://seom.org/images/Libro_Cancer_hereditario_2019.pdf

[9] Stanich PP, Pearlman R, Hinton A, et al. Prevalence of germline mutations in polyposis and colorectal cancer-associated genes in patients with multiple colorectal polyps. Clin Gastroenterol Hepatol. 2019;17:2008–15. 30557735 10.1016/j.cgh.2018.12.008

[10] Servicio Madrileño de Salud. Plan de Cáncer Familiar. Comunidad de Madrid. Madrid, 2015. [cited March 26, 2024] Available from: https://www.comunidad.madrid/servicios/salud/programa-cancer-familiar#panel-43292

[11] Fernandez-Felix BM, García-Esquinas E, Muriel A, et al. Bootstrap internal validation command for predictive logistic regression models. Stand Genomic Sci. 2021;21:498–509.

[12] Consejería de Sanidad Servicio Madrileño de Salud. Prevecolon: prevención del cáncer de colon y recto [cited April 02, 2024] Available from: https://www.comunidad.madrid/servicios/salud/prevecolon-prevencion-cancer-colon-recto

[13] Oficina regional de coordinación oncológica. Dirección general de coordinación de la asistencia sanitaria. Consejería de Sanidad. Memoria del programa de cribado de cáncer de colon y recto PREVECOLON. 2017. [cited April 02, 2024] Available from: https://www.comunidad.madrid/sites/default/files/doc/sanidad/asis/memoria_prevecolon_2017.pdf

[14] Consejería de Sanidad Servicio Madrileño de Salud. Observatorio de resultados del Servicio Madrileño de Salud. Datos generales - Detección precoz de cáncer [cited April 15, 2024]. Available from: http://observatorioresultados.sanidadmadrid.org/HospitalesDatosGeneralesTabla.aspx?ID=106

[15] Kastrinos F, Samadder NJ, Burt RW. Use of family history and genetic testing to determine risk of colorectal cancer. Gastroenterology. 2020;158:389–403. 31759928 10.1053/j.gastro.2019.11.029

[16] Armelao F, de Pretis G. Familial colorectal cancer: a review. World J Gastroenterol. 2014;20:9292–8. 25071323 10.3748/wjg.v20.i28.9292 PMC4110560

[17] Basso G, Bianchi P, Malesci A, et al. Hereditary or sporadic polyposis syndromes. Best Pract Res Clin Gastroenterol. 2017;31:409–17. 28842050 10.1016/j.bpg.2017.05.011

[18] Cubiella J, Marzo-Castillejo M, Mascort-Roca JJ, et al; Sociedad Española de Medicina de Familia y Comunitaria y Asociación Española de Gastroenterología. Clinical practice guideline. Diagnosis and prevention of colorectal cancer. 2018 Update. Gastroenterol Hepatol 2018;41:585-96 10.1016/j.gastrohep.2018.07.012 30245076

[19] Mak S, Alexander JL, Clark SK, Hawkins M, et al. The diagnostic yield of genetic testing in patients with multiple colorectal adenomas: a specialist center cohort study. Clin Transl Gastroenterol. 2024;15: e00645. 37856205 10.14309/ctg.0000000000000645 PMC10810582

[20] Aretz S, Tricarico R, Papi L, et al. MUTYH-associated polyposis (MAP): evidence for the origin of the common European mutations p.Tyr179Cys and p.Gly396Asp by founder events. Eur J Hum Genet. 2014;22:923–9 10.1038/ejhg.2012.309 PMC4060104 23361220

[21] Barreiro RAS, Sabbaga J, Rossi BM, et al. Monoallelic deleterious MUTYH germline variants as a driver for tumorigenesis. J Pathol. 2022;256:214–22. 34816434 10.1002/path.5829

[22] Paller CJ, Tukachinsky H, Maertens A, et al. Pan-cancer interrogation of MUTYH Variants reveals biallelic inactivation and defective base excision repair across a spectrum of solid tumors. JCO Precis Oncol. 2024;8: e2300251. 38394468 10.1200/PO.23.00251 PMC10901435

[23] Qumseya BJ, Coe S, Wallace MB. The effect of polyp location and patient gender on the presence of dysplasia in colonic polyps. Clin Transl Gastroenterol. 2012;3: e20. 23238292 10.1038/ctg.2012.14 PMC3412677

[24] Kazem-Shahmoradi M, Soleimaninejad M, Sharifian M. Evaluation of colonoscopy data for colorectal polyps and associated histopathological findings. Ann Med Surg (Lond). 2020;57:7–11. 32685144 10.1016/j.amsu.2020.07.010 PMC7358369

[25] Kalady MF, Church JM. Prophylactic colectomy: Rationale, indications, and approach. J Surg Oncol. 2015;111:112–7. 25418116 10.1002/jso.23820

[26] Vogelsang HE. Prophylactic surgery and extended oncologic radicality in gastric and colorectal hereditary Cancer syndromes. Visc Med. 2019;35:231–9. 31602384 10.1159/000501919 PMC6738259

[27] Cross AJ, Robbins EC, Pack K, et al. Colorectal cancer risk following polypectomy in a multicentre, retrospective, cohort study: an evaluation of the 2020 UK post-polypectomy surveillance guidelines. Gut. 2021;70:2307–20. 33674342 10.1136/gutjnl-2020-323411 PMC8588296

[28] Yoshida N, Ishikawa H, Eguchi H, et al. Promotion effects of smoking in polyp development in monozygotic twins with atypical colorectal polyposis. Case Rep Gastroenterol. 2022;16:375–81. 35949244 10.1159/000524944 PMC9247489

[29] Botteri E, Iodice S, Raimondi S, et al. Cigarette smoking and adenomatous polyps: a meta-analysis. Gastroenterology. 2008;134:388–95. 18242207 10.1053/j.gastro.2007.11.007

[30] PDQ Cancer Genetics Editorial Board. Genetics of Colorectal Cancer (PDQ®): Health Professional Version. 2024 Feb 2. In: PDQ Cancer Information Summaries. Bethesda (MD): National Cancer Institute (US); 2002

[31] Terlouw D, Suerink M, Singh SS, et al. Declining detection rates for APC and biallelic MUTYH variants in polyposis patients, implications for DNA testing policy. Eur J Hum Genet. 2020;28:222–30. 31527860 10.1038/s41431-019-0509-z PMC6974599

[32] Valle L, Vilar E, Tavtigian SV, et al. Genetic predisposition to colorectal cancer: syndromes, genes, classification of genetic variants and implications for precision medicine. J Pathol. 2019;247:574–88. 30584801 10.1002/path.5229 PMC6747691

[33] Schubert SA, Morreau H, de Miranda NFCC, van Wezel T. The missing heritability of familial colorectal cancer. Mutagenesis. 2020;35:221–31. 31605533 10.1093/mutage/gez027 PMC7352099

[34] Valle L, de Voer RM, Goldberg Y, et al. Update on genetic predisposition to colorectal cancer and polyposis. Mol Aspects Med. 2019;69:10–26. 30862463 10.1016/j.mam.2019.03.001

[35] Fostira F, Kontopodis E, Apostolou P, et al. Extending the clinical phenotype associated with biallelic NTHL1 germline mutations. Clin Genet. 2018;94:588–9. 30248171 10.1111/cge.13444

