# Supplementary material for: Optimizing genetic testing strategy for suspected attenuated adenomatous polyposis: effective solutions in public health systems
Source: Clin Transl Oncol. 2024 Dec 11;27(6):2710–8. doi: 10.1007/s12094-024-03811-y (PMC12084234; doi:10.1007/s12094-024-03811-y)
Supplement: Supplementary file 1 — Supplementary file1 (PPTX 1086 KB) [file 12094_2024_3811_MOESM1_ESM.pptx]

## Slide 1
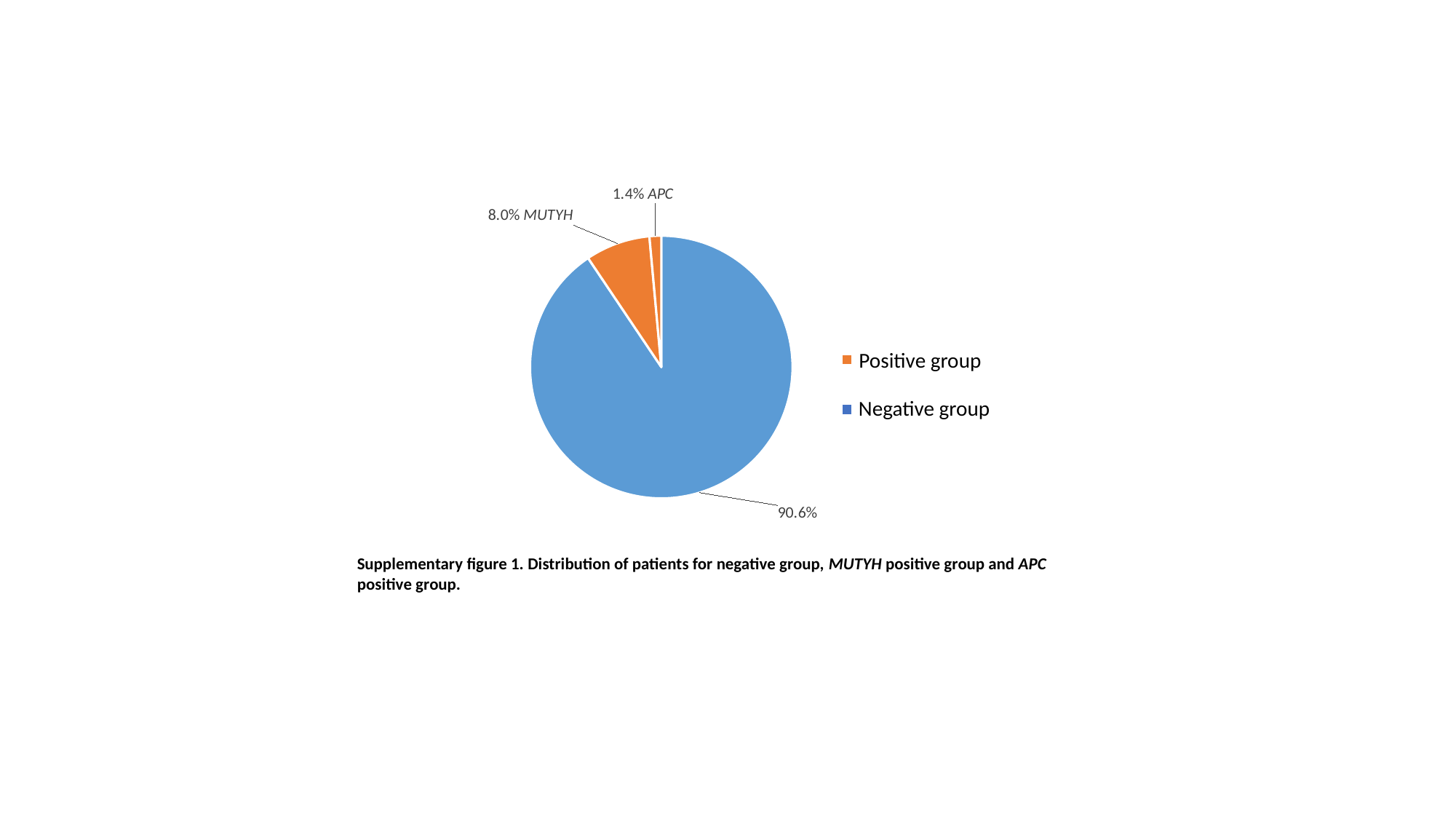

### Chart
| Category | |
|---|---|
| Negativos | 90.57971014492753 |
| MUTYH | 7.971014492753623 |
| APC | 1.4492753623188406 |
Positive group
Negative group
Supplementary figure 1. Distribution of patients for negative group, MUTYH positive group and APC positive group.

## Slide 2
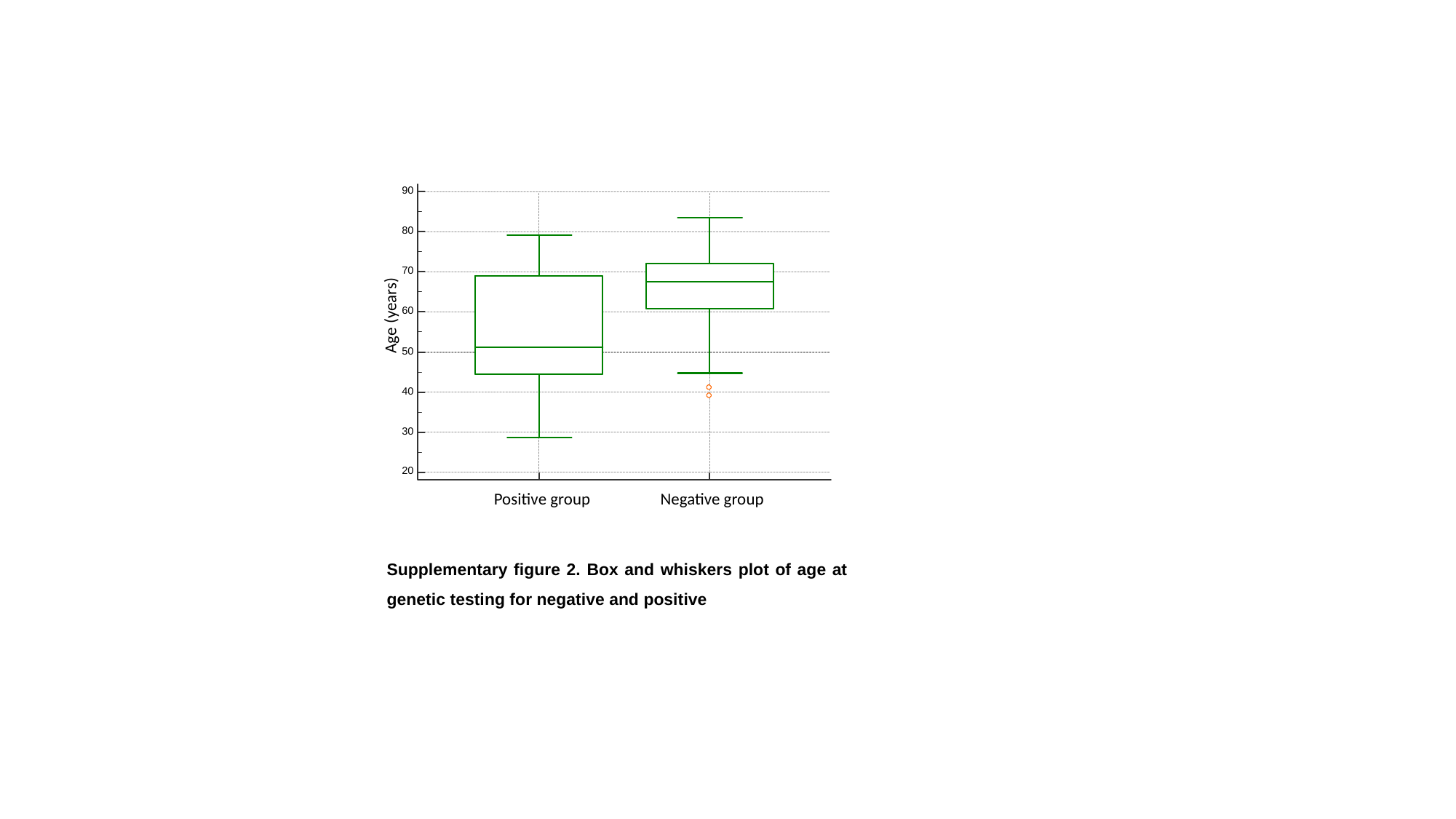

Age (years)
Positive group
Negative group
Supplementary figure 2. Box and whiskers plot of age at genetic testing for negative and positive

## Slide 3
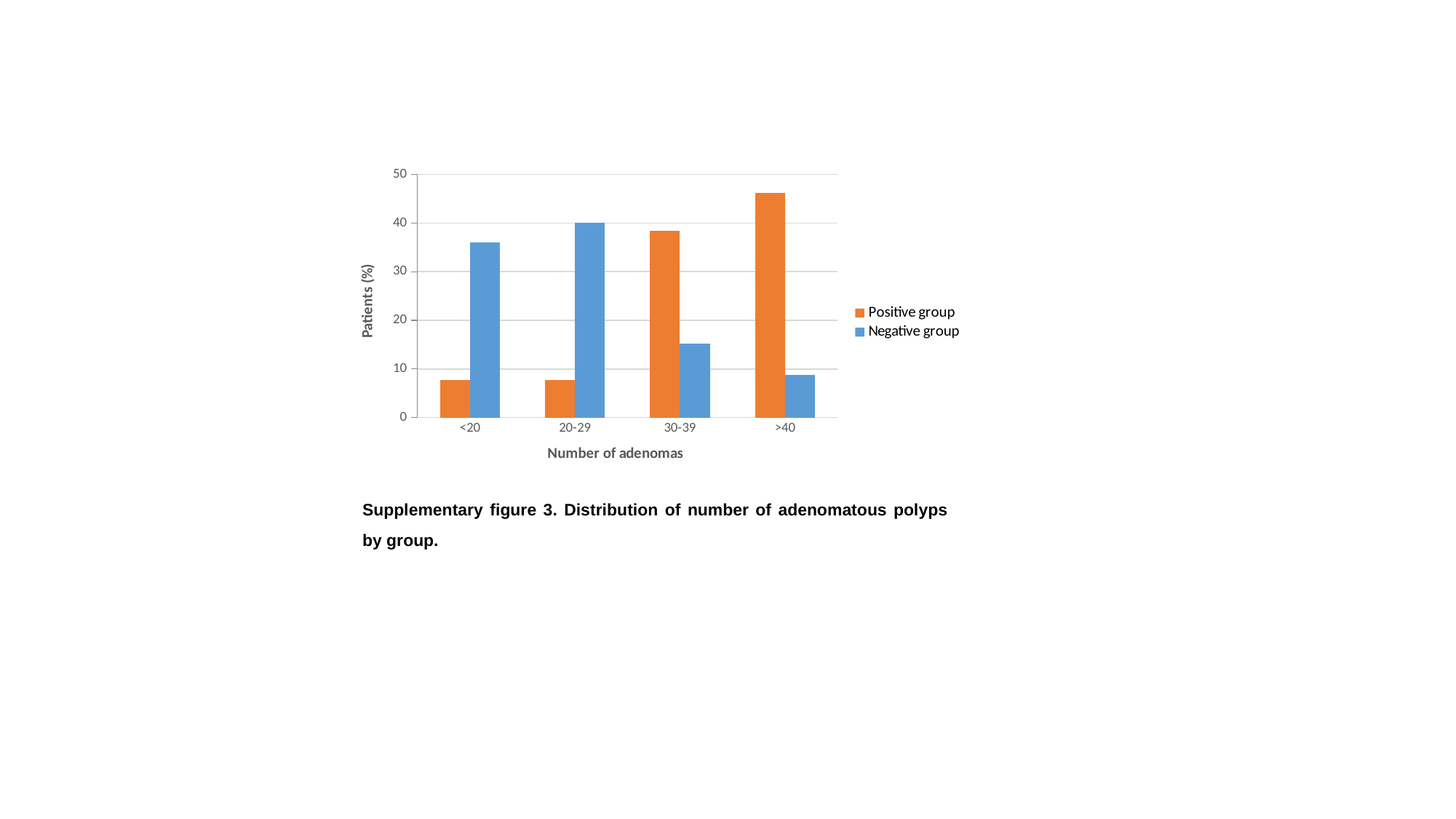

### Chart
| Category | | |
|---|---|---|
| <20 | 7.6923076923076925 | 36.0 |
| 20-29 | 7.6923076923076925 | 40.0 |
| 30-39 | 38.46153846153846 | 15.2 |
| >40 | 46.15384615384615 | 8.8 |Supplementary figure 3. Distribution of number of adenomatous polyps by group.

## Slide 4
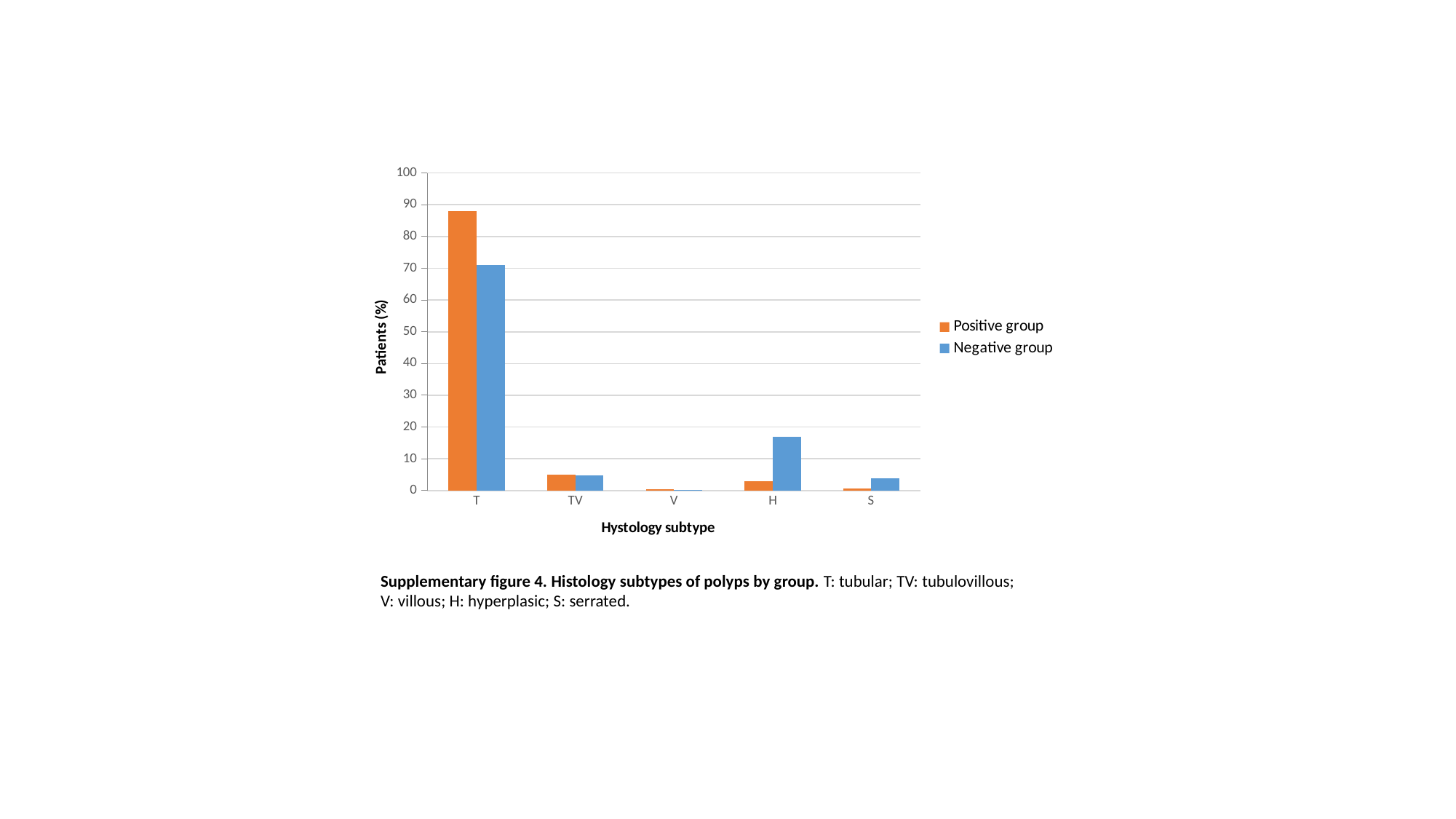

### Chart
| Category | | |
|---|---|---|
| T | 87.87446504992867 | 71.09905020352781 |
| TV | 4.992867332382311 | 4.85753052917232 |
| V | 0.42796005706134094 | 0.18995929443690637 |
| H | 2.8530670470756063 | 16.987788331071915 |
| S | 0.5706134094151213 | 3.8805970149253732 |Supplementary figure 4. Histology subtypes of polyps by group. T: tubular; TV: tubulovillous; V: villous; H: hyperplasic; S: serrated.

## Slide 5
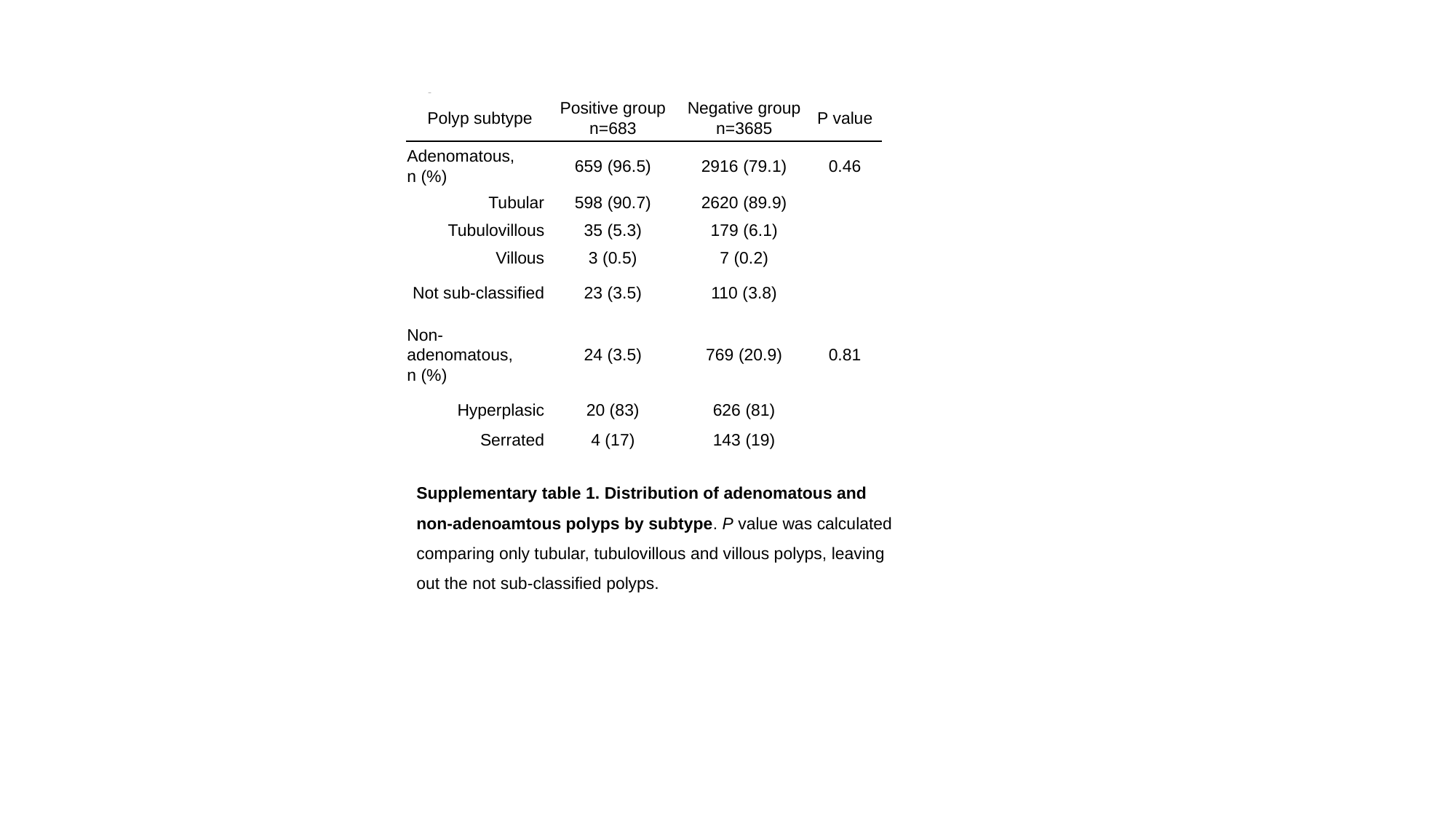

### Chart
| Category | Positives | Negatives |
|---|---|---|
| ≤30 | 7.6923076923076925 | 0.0 |
| 31-40 | 7.6923076923076925 | 0.8 |
| 41-50 | 30.76923076923077 | 4.8 |
| 51-60 | 15.384615384615385 | 20.0 |
| 61-70 | 15.384615384615385 | 44.0 |
| >70 | 23.076923076923077 | 30.4 |
### Chart
| Category | Positives | Negatives |
|---|---|---|
| ≤30 | 7.6923076923076925 | 0.0 |
| 31-40 | 7.6923076923076925 | 0.8 |
| 41-50 | 30.76923076923077 | 4.8 |
| 51-60 | 15.384615384615385 | 20.0 |
| 61-70 | 15.384615384615385 | 44.0 |
| >70 | 23.076923076923077 | 30.4 || Polyp subtype | Positive group n=683 | Negative group n=3685 | P value |
| --- | --- | --- | --- |
| Adenomatous, n (%) | 659 (96.5) | 2916 (79.1) | 0.46 |
| Tubular | 598 (90.7) | 2620 (89.9) | |
| Tubulovillous | 35 (5.3) | 179 (6.1) | |
| Villous | 3 (0.5) | 7 (0.2) | |
| Not sub-classified | 23 (3.5) | 110 (3.8) | |
| Non-adenomatous, n (%) | 24 (3.5) | 769 (20.9) | 0.81 |
| Hyperplasic | 20 (83) | 626 (81) | |
| Serrated | 4 (17) | 143 (19) | |
Supplementary table 1. Distribution of adenomatous and non-adenoamtous polyps by subtype. P value was calculated comparing only tubular, tubulovillous and villous polyps, leaving out the not sub-classified polyps.

## Slide 6
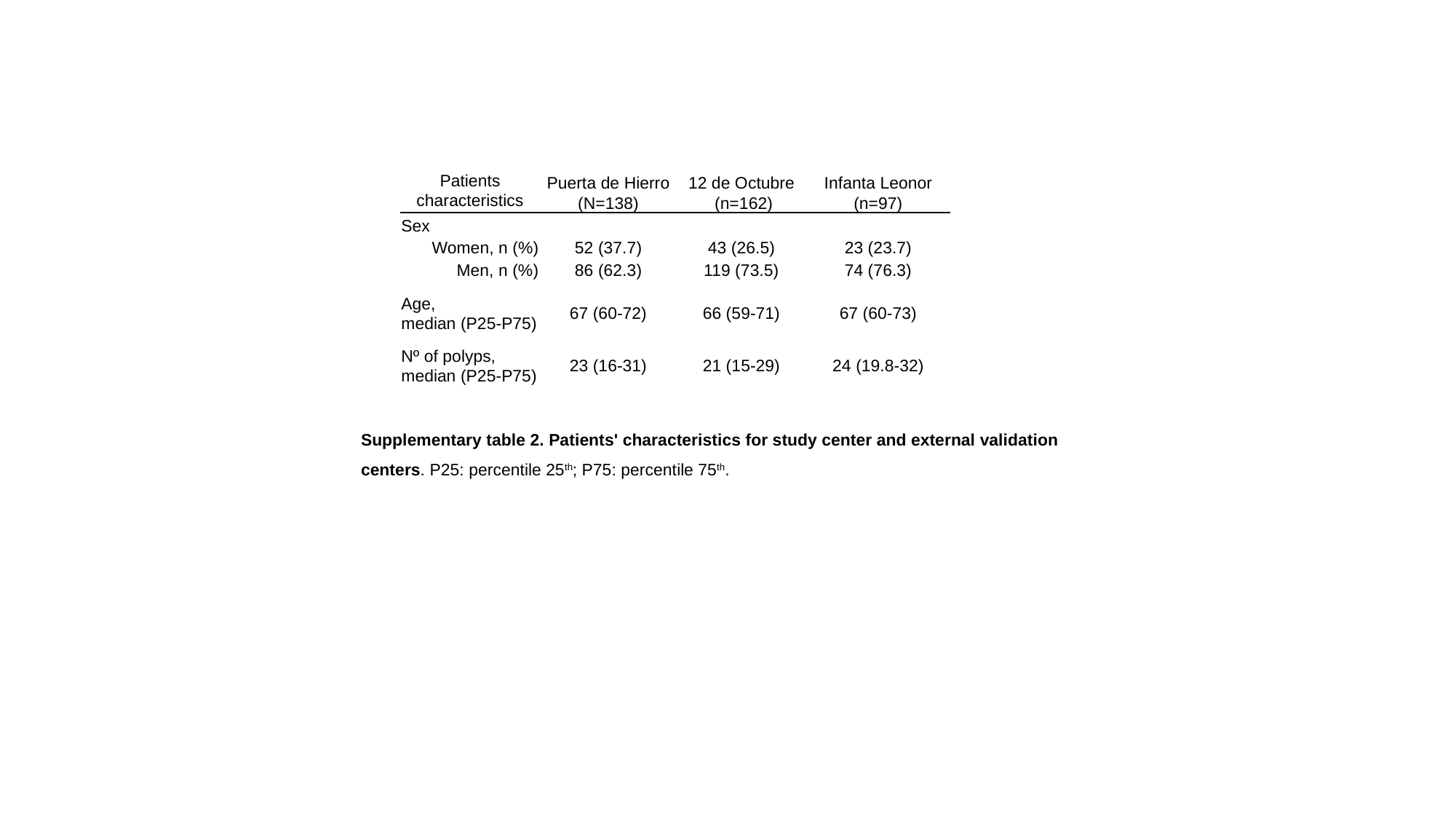

| Patients characteristics | Puerta de Hierro (N=138) | 12 de Octubre (n=162) | Infanta Leonor (n=97) |
| --- | --- | --- | --- |
| Sex | | | |
| Women, n (%) | 52 (37.7) | 43 (26.5) | 23 (23.7) |
| Men, n (%) | 86 (62.3) | 119 (73.5) | 74 (76.3) |
| Age, median (P25-P75) | 67 (60-72) | 66 (59-71) | 67 (60-73) |
| Nº of polyps, median (P25-P75) | 23 (16-31) | 21 (15-29) | 24 (19.8-32) |
Supplementary table 2. Patients' characteristics for study center and external validation centers. P25: percentile 25th; P75: percentile 75th.
